# Endocrine Disruptors: Bisphenol A and the Brain

**Published:** 2006-04

**Authors:** Julia R. Barrett

Estrogens are known to trigger rapid cellular responses, including hormone secretion and cell permeability changes, in tissues as diverse as the pancreas, the pituitary gland, and the brain. Two studies published in the December 2005 issue of *Endocrinology* now present an intricate picture of how estradiol, the body’s primary endogenous estrogen, acts in the cerebellum, with one study building on the other and including another layer of complexity: the addition of the xenoestrogen bisphenol A to the system.

The studies, conducted by researchers led by Scott Belcher at the University of Cincinnati College of Medicine, investigated an estrogen-mediated extracellular signal–regulated kinase system in developing rat cerebella neurons. The first study, conducted *in vitro*, identified the individual steps in a cascade of cellular responses triggered by estradiol. The second study focused on this response following injection of estradiol and bisphenol A, alone and in combination, into rat cerebella. Effects were seen at very low doses of 10^−12^ to 10^−10^ moles per liter (M) and at higher doses of 10^−7^ to 10^−6^ M, but not at intermediate doses of 10^−9^ to 10^−8^ M. Paradoxically, when bisphenol A was injected alone, it mimicked estradiol; when injected with estradiol, however, it blocked estradiol action.

Bisphenol A is a known endocrine-active chemical. Low-level human exposure is widespread due to the chemical’s presence in polycarbonate plastic and epoxy resins, but understanding long-term consequences of exposure will be challenging. As illustrated in the *Endocrinology* papers, bisphenol A exerts an effect through a complex system at a concentration range that has not been evaluated in traditional risk assessment.

“It is a fundamental part of endocrinology, and it is beautifully demonstrated [in these papers], that stimulation at [the cerebellar] cell surface receptor is able to cause effects at doses below a part per trillion,” says Frederick vom Saal, a professor of biology at the University of Missouri–Columbia. Not only are the doses many magnitudes lower than those considered in classic high-dose toxicity studies, but at extremely low doses both estradiol and bisphenol A demonstrate a response that disappears as the dose increases. “This absolutely challenges the fundamental assumption of risk assessment that once you start increasing dose you always see an increase in response,” says vom Saal.

According to Belcher, an assistant professor of pharmacology and cell biophysics, labeling the effects observed in his group’s studies as harmful or negative is not possible. “With the way it’s been looked at, you can’t say whether the observed actions are safe or harmful, but it is clear that the issue needs to be looked at more carefully and seriously,” he says.

Although the plastics manufacturing industry, represented by the American Plastics Council in Arlington, Virginia, generally questions several aspects of bisphenol A research, they do agree that translating findings such as those in the *Endocrinology* papers to the sphere of risk assessment won’t be easy. “It’s not so straightforward to figure out what the results mean for human health, even if you take the results that are published at face value,” says Steve Hentges, executive director of the American Plastics Council polycarbonate business unit. “Even to develop a testable hypothesis is not very simple at all. It’s very complex systems that they’re looking at. The mechanism is a long way from any kind of an adverse effect.”

Belcher, too, believes that extending his work into risk assessment is premature. “You can’t make a conclusion whether bisphenol A is going to be safe or harmful with current risk assessment models at these low doses,” he says. Further, as shown by the paradoxical reaction to bisphenol A, responses to an estrogenic compound can depend on what else is in the system.

## Figures and Tables

**Figure:**
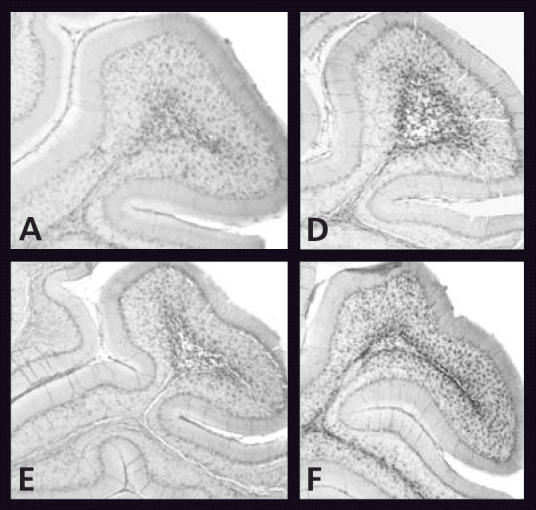
Are U surprised? Compared to the control (A), bisphenol A exhibits a U-shaped dose–response curve in the rat cerebellum, increasing immunopositive cell numbers at low and high doses (D, F, respectively), but not intermediate doses (E).

